# Evaluation of the first open-access hepatitis B and safe injection online training course for health professionals in China

**DOI:** 10.1186/s12909-016-0608-2

**Published:** 2016-03-08

**Authors:** Jing Wang, Qiming Feng, Andrew Tam, Tong Sun, Peijing Zhou, Samuel So

**Affiliations:** Asian Liver Center at Stanford University, 780 Welch Road, CJ130, 94304 Palo Alto, CA USA; School of Public Health, Guangxi Medical University, No.22, Shuangyong Road, Nanning City, Guangxi Province China; Shandong Provincial Center for Disease Control and Prevention, No. 16992, Jingshi Road, Jinan City, Shandong Province China

**Keywords:** Hepatitis B, Internet based education, Open-access education, Online learning, Continuing medical education, Online learning evaluation

## Abstract

**Background:**

Despite the high prevalence of chronic hepatitis B virus (HBV) infection in China, HBV infection prevention and long-term care knowledge of health professionals is inadequate. To address this knowledge gap, we developed an open-access evidence-based online training course, "KnowHBV", to train health professionals on prevention of HBV transmission and safe injections. We conducted an evaluation of the course with health professionals in China to examine its effectiveness in improving knowledge and learner’s satisfaction of the course.

**Methods:**

Between July and December 2011, 1015 health professionals from selected hospitals and disease control institutions of Shandong province registered for the course and 932 (92 %) completed the three-module course. Participants’ demographic information, pre- and post-course knowledge test results and learner’s feedback were collected through the course website.

**Results:**

Pre-course knowledge assessment confirmed gaps in HBV transmission routes, prevention and long-term care knowledge. Only 50.4 % of participants correctly identified all of the transmission routes of HBV, and only 40.7 % recognized all of the recommended tests to monitor chronically infected persons. The number of participants that answered all six multi-part multiple-choice knowledge questions correctly increased from 183 (19.7 %) before taking the course to 395 (42.4 %) on their first attempt upon completion of the course. Over 90 % of the 898 participants who completed the learner-feedback questionnaire rated the course as ‘good’ or ‘very good’; over 94 % found the course instructional design helpful; 57.5 %, 65.7 % and 68.5 % reported that half or more than half of the course content in modules 1, 2 and 3 respectively provided new information; and 93.2 % of the participants indicated they preferred the online learning over traditional face-to-face classroom learning.

**Conclusions:**

The "KnowHBV" online training course appears to be an effective online training tool to improve HBV prevention and care knowledge of the health professionals in China.

## Background

Chronic hepatitis B virus (HBV) infection is a major public health problem in China, but it is entirely preventable through vaccination and safe injection practices. Monitoring of people living with chronic HBV infection and antiviral drug treatment when indicated would reduce the risk of disease progression and deaths from cirrhosis and liver cancer [1]. China has the greatest burden of chronic HBV infection in the world, with an estimated 93 million people under age 60 years living with chronic HBV infection in 2006, representing approximately 7.2 % of the country’s total population [2]. Liver cancer and liver disease caused by acute and chronic HBV infection accounted for 263,000-300,000 deaths in China and made up more than 40 % of the worldwide deaths associated with HBV infection per year, and the number continues to increase in recent years [3–5]. According to a recent study in Shandong province, treatment of chronic HBV infection imposed considerable economic burden on the affected families. As high as 40–150 % of the average annual household income was spent on out-of-pocket costs relating to treatment and care, even in patients with health insurance coverage [6].

Despite the high burden of HBV infection in China, knowledge and practice among public health and healthcare professionals on HBV transmission routes, prevention and the long-term care of chronically infected persons have been shown to be lacking [7, 8]. This was especially true for primary healthcare providers working at township, community and village hospitals [9]. A survey conducted in 2011 in a county in Gansu province found that 78 % of the township and village doctors believed that hepatitis B could be transmitted through sharing of food and cups [10]. Misconception about HBV’s transmission through casual contacts and sharing food among health care providers had contributed to widespread discrimination against people who were chronically infected in China [11].

According to World Health Organization (WHO), unsafe injection practice is a major cause of hospital-acquired HBV infection in developing countries [12]. Unnecessary and unsafe injections in the healthcare settings remain common in the rural areas in China. Prescription injection rate at health care facilities in rural areas at nine central and western provinces or cities was 25.8–62.2 % [13]. A survey conducted in Chongqing city discovered that 77 % of the health facilities have at least one unsafe injection practice [14], and an outbreak of hepatitis C infection in 2011 due to reusing needles and syringes at a rural village clinic near Henan and Anhui provinces showed there is an urgent need to improve safe injection practices to prevent the transmission of blood-borne diseases among rural healthcare providers in China [15]. Yet, there has been no large scale evidence-based training in injection safety for the vast number of healthcare practitioners working at community and rural areas in China.

Rapid adoption of the internet by the Chinese population and healthcare facilities opened up an untapped opportunity to rapidly improve the training of health care workers across even the remote parts of China. The China Internet Network Information Center estimated that by June 2013, 668 million people in China (49 %) were internet users [16]. Following the completion of the world’s largest infectious disease reporting system, almost all hospitals and clinics above township level across China have internet access to report electronically known and unknown infectious diseases [17].

To address the training needs of health workers on hepatitis B prevention and care coupled with the growing popularity and access to internet in China, the Asian Liver Center at Stanford University collaborated with the Shandong Centers for Disease Control and Prevention (CDC) to develop an evidence-based, free online training course in Chinese –“KnowHBV” (www.knowhbv.org). The course’s target audiences were the health professionals in China, which we defined as all health care providers including western medicine and traditional Chinese medicine doctors and nurses, as well as public health professionals. Our course content was specifically tailored for primary care doctors and nurses currently practicing at township, community and village hospitals and clinics. To our best knowledge, this is the first open-access online hepatitis B and safe injection training course in Chinese.

To assess the course’s effectiveness in improving knowledge and user’s acceptance of online training as a means of improving hepatitis B prevention and care knowledge, we conducted an evaluation of the course with health professionals in Shandong Province, China.

## Methods

### Evaluation structure

**Fig. 1:**
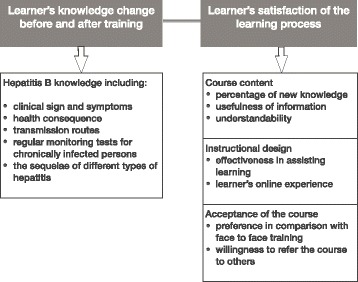
Evaluation framework of the KnowHBV online training course

The evaluation structure of the course was based on the first two levels of Kirkpatrick's Four Level Outcome Evaluation Model, namely evaluating the learner’s gain of knowledge and skill and learner’s satisfaction of the learning process, which are recognized as the most essential steps to assess user’s online learning process in terms of course design and delivery, knowledge and skill change [18] (Fig. 1).

Data were obtained online through six multi-part, multiple choice pre- and post-course test questions, and a learner-feedback questionnaire. The pre- and post-course knowledge tests consisted of the same multi-part questions to assess participants’ hepatitis B knowledge. The learner-feedback questionnaire invited participants to rate the course on a 5-point Likert scale from 1 as the lowest to 5 as the highest rating on the following categories: course content, instructional design and acceptance of the course. In addition, the questionnaire included three open-ended questions in order for us to gain more insight into the different aspects of learners’ experience with our course that had not already been addressed in the other sections of the questionnaire.

The web pages and the training course were all in Chinese. To access the training course, new participants needed to register for a free user account at the course website with a personal or work email address. During the registration, participants were requested to provide information, such as work location, profession, education level, and work title. The course was structured so that the participants were only permitted to take the training course in the following order: the pre-course hepatitis B knowledge test, the three training modules and their respective 3–5 questions quiz, the post-course knowledge test, and the learner-feedback questionnaire. Participants who completed all the steps and answered all the post-course survey questions correctly would be able to print out a certificate of completion. No written consent forms were obtained in this online study from the participating health professionals. An introduction of the course was presented on the first page of the website with a continue button for the participants to get to the training page.

### Course content and instructional design

The course content was tailored for healthcare workers and public health professionals in China and covered knowledge gaps and misconceptions of HBV transmission routes identified by published research studies [7–9]. The evidence-based course included the latest recommendations from WHO, U.S. CDC, Chinese CDC and professional practice guidelines adopted in the U.S. and in China. The course consisted of three modules: 1) public health impact of hepatitis B in China, HBV facts and hepatitis B vaccination; 2) safe injection practices at the healthcare settings to prevent transmission of hepatitis B and other blood borne pathogens; and 3) physician-patient communication, interpretation of hepatitis B test results and long-term care of chronically infected persons. The course was pilot tested with local Chinese speakers in the U.S. before putting it online for evaluation in China.

We used the open-source Moodle Course Management System to provide a platform to host the training modules, survey tools, and to regulate the learning process of participants. The online modules featured streamlined slides with Chinese voice-over and subtitles. Moreover, the course’s instructional design aimed at increasing knowledge retention, using information repetition, embedded pictures and videos, case studies/scenarios, and interactive quizzes with knowledge reinforcement for correct answers. The content navigation bar enabled users to access different slides easily. Also, there was a reference list linking users to free abstracts or full text articles.

### Participant recruitment

We collaborated with the Shandong Provincial Health Department, Shandong CDC, and Shandong University to recruit health professionals and students from selected hospitals and medical schools in Shandong. One provincial level hospital, one regional level hospital, one county level hospital and all township hospitals in two counties, as well as public health professionals from corresponding health departments and CDCs participated in our course evaluation. The total number of the employees within these institutions was 3399. Through the use of official announcements from Shandong CDC, onsite electronic billboard, and wall posters, employees from the selected institutions were encouraged to take the online course. In addition, participants were informed that Shandong health department would offer three Continuing Medical Education (CME) credits for those who completed the course and received the certificate of completion. Shandong University also encouraged students from its public health and nursing schools to take the online training.

### Data collection and analysis

Data were collected online through Moodle’s back-end data collecting and reporting system. During the data cleaning process, we excluded participants that registered from outside of the Shandong Province and those that did not complete the required course components (pre-course survey, the three training modules, and post-course survey). Furthermore, we eliminated multiple registrations from the same user based on name and demographic information provided and kept only the earliest entries. Since participants were allowed to take the post course knowledge tests multiple times and must eventually answer all the questions correctly at the post course test in order to receive a course completion certificate, we only included responses from participants’ first attempt upon completion of the course into the database for analysis.

We applied the *McNemar’s test* and the *paired t-test* to compare changes in knowledge in the pre- and post-course tests. Descriptive statistical analysis was used to illustrate participants’ ratings on different aspects of the course. All analyses were performed using Microsoft Excel and Stata-IC version 12.

The study was approved by the Ethical Review Board for Human Related Biomedical and Preventive Medical Research at the Center for Disease Control and Prevention of Shandong Province, China. Reference number is (2011–8).

## Results

### Characteristics of the participants

Between July 1 and December 1, 2011, 1,015 participants comprising 812 health professionals and 203 students from Shandong Province registered for the course. Among the registrants, 932 (91.8 %) completed the training course and the pre- and post-course tests. They included 195 (20.9 %) doctors, 170 (18.3 %) nurses, 333 (35.7 %) disease control personnel, 203 (21.8 %) students from public health and nursing schools, and 31 (3.3 %) listed as other health professionals (technicians, etc.). Three hundred and twenty-two (34.5 %) registrants reported they worked in the hospitals and CDC at the provincial level, 166 (17.8 %) at the regional level, 116 (12.5 %) at the county level, and 119 (12.8 %) at the township level. Among the remaining 209 registrants, 203 (21.8 %) identified themselves as current university students. Six hundred and seventy-three (72.2 %) participants reported to have formal medical education degree in China—Bachelor of Medicine.

### Learner’s knowledge change

The pre-course test results showed that among the 932 participants, only 183 (19.6 %) answered all six multi-part questions correctly. Only 470 (50.4 %) participants identified all the hepatitis B transmission routes correctly, and only 379 (40.7 %) recognized all the recommended tests to monitor chronically infected persons. Percentage of participants who answered all six questions correctly increased from 19.6 to 42.4 % upon completion of the course. For each of the six questions, the percentage of participants who answered the questions correctly increased significantly upon completion of the course modules (all *P* < 0.001) (Table 1).

**Table 1:** Comparison of participants’ hepatitis B knowledge before and after online training

Questions	Pre-course correct answer	Post-course correct answer	McNemar’s test	*P* value
	*n* (%)	*n* (%)		
Q1. Symptoms of chronic hepatitis B (single choice) a. Always has symptoms b. Most of time is symptomatic **c. Usually is asymptomatic**	760 (81.6)	896 (96.1)	120.10	<0.001
Q2. Complications of chronic hepatitis B (single choice) a. Liver damage and cirrhosis b. Liver cancer c. Premature death **d. All of the above**	773 (82.9)	859 (92.2)	57.78	<0.001
Q3. Transmission routes of hepatitis B (multiple choices) a. Contaminated food and water **b. Contaminated blood** **c. Reuse of needle and syringes** **d. Unprotected sex with infected person** **e. Infected mother infect the newborn during delivery** f. Shake hands with infected persons g. Sneezing or coughing h. Share food and utensils with infected person	470 (50.4)	711 (76.3)	186.76	<0.001
Q4. Ways to prevent the transmission of hepatitis B (multiple choices) a. Make sure food is well cooked **b. Get hepatitis B vaccination** **c. Avoid reuse of needles and syringes** d. Avoid drinking contaminated water	542 (58.2)	773 (82.9)	180.88	<0.001
Q5. Regular checkup tests for persons with chronic hepatitis B (multiple choices) a. AFP test **b. ALT test** **c. Abdominal ultrasound** d. No need to test when there is no symptoms	379 (40.7)	742 (79.6)	331.91	<0.001
Q6. Types of hepatitis that can lead to chronic infection (multiple choices) a. Hepatitis A **b. Hepatitis B** **c. Hepatitis C**	500 (53.7)	602 (64.6)	51.00	<0.001
All 6 questions	183 (19.6)	395 (42.4)	187.27	<0.001

Answers in bold were correct answers

Participants were assigned one point for answering each of the questions correctly. Out of the total score of six, participants’ mean scores on the pre- and post-course tests were 3.67 (95 % CI, 3.57, 3.78) and 4.92 (95 % CI, 4.84, 5.00) respectively (*P* < 0.001). Significant increases were demonstrated across all professional categories and work levels using the paired *t*-test (Table 2).

**Table 2:** Comparison of mean scores by profession and working level on the pre- and post- course tests

		Pre-course x- ± s	Post-course x- ± s	*t*-test	*P* value
*N* = 932		3.67 ± 1.69	4.92 ± 1.22	24.90	<0.001
Profession	Doctor	4.09 ± 1.44	4.98 ± 1.08	8.97	<0.001
	Nurse	3.30 ± 1.72	4.41 ± 1.39	9.68	<0.001
	Disease Control	4.12 ± 1.70	5.12 ± 1.18	12.43	<0.001
	Student	3.02 ± 1.52	4.99 ± 1.15	18.79	<0.001
	Other	2.61 ± 1.54	4.61 ± 1.15	7.40	<0.001
Working	Provincial	3.85 ± 1.61	4.75 ± 1.33	11.68	<0.001
Level	City	4.01 ± 1.76	5.16 ± 1.17	9.62	<0.001
	County	3.73 ± 1.72	4.85 ± 1.25	7.90	<0.001
	Township	3.91 ± 1.68	4.98 ± 0.98	7.96	<0.001
	Other^a^	2.98 ± 1.54	4.97 ± 1.15	19.42	<0.001

^a^Included all students from Shandong University

### Learner’s satisfaction of the course

**Fig. 2:**
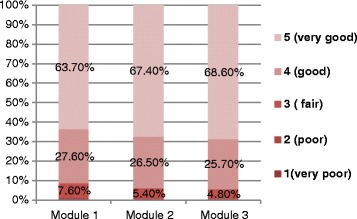
Distribution of participants’ rating for quality of three course modules. This means 91.3, 93.9 and 94.3 % of the participants rated the quality of module 1, module 2 and module 3 as “good” or “very good”; only 1.2, 0.8 and 0.9 % rated module 1, module 2 and module 3 respectively as “poor” or “very poor”

Of the 1015 participants that registered for the course, 898 (88.5 %) completed the feedback questionnaire besides the pre- and post-knowledge tests. Among those who completed the feedback questionnaire, 91.3, 93.9 and 94.3 % rated the overall quality of the modules 1, 2, and 3 as “good” or “very good” (Fig. 2).

#### Course content

The course content was rated in the following three aspects: percentage of new knowledge for the learners, usefulness for daily practice and understandability. These ratings from users helped us determine the appropriateness of the course content for the primary healthcare workers.

**Fig. 3:**
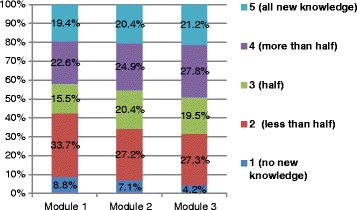
Distribution of participants’ rating of new knowledge in three course modules. This means 57.5. 65.7 and 68.5 % of the participants responded that module1, module 2 and module 3 respectively have at least half of the information that was previously unknown to them; 8.8, 7.1 and 4.2 % stated that there was no new knowledge in the course

Results showed that 57.5, 65.7 and 68.5 % of the participants responded that modules 1, 2 and 3 respectively have at least half of the information that was previously unknown to them. Only 8.8, 7.1 and 4.2 % stated that there was no new knowledge in the course (Fig. 3). As many as 224 (25.0 %) participants agreed and 635 (70.7 %) participants strongly agreed that the course content was useful for their daily practice. Two hundred and forty-five (27.3 %) participants agreed and 614 (68.4 %) strongly agreed that the course content was easy to understand.

#### Instructional design

The interactive quiz, animation, case scenario, and voice-over in Chinese received high ratings as “very helpful” in assisting the course learning; the reference list linking to full articles (in Chinese and English) and the Chinese transcript on the slides got relatively lower rating in assisting course learning in comparison to other features (Table 3).

**Table 3:** Distribution of participants’ rating of course instructional design components

Instructional design component	1 (not helpful)	2 (less helpful)	3 (somewhat helpful)	4 (helpful)	5 (very helpful)
Transcript	13(1.5)	25(2.8)	112(12.5)	286(31.9)	462(51.5)
Voice-over	7(0.8)	17(1.9)	83(9.2)	283(31.5)	508(56.6)
Reference	21(2.3)	29(3.2)	128(14.3)	280(31.2)	440(49.0)
Pictures	8(0.9)	16(1.8)	98(10.9)	278(31.0)	498(55.5)
Animation & video	4(0.5)	9(1.0)	97(10.8)	262(29.2)	526(58.6)
Case scenario	3(0.3)	12(1.3)	85(9.5)	274(30.5)	524(58.4)
Interactive Quizzes	5(0.6)	11(1.2)	73(8.1)	255(28.4)	554(61.7)

For user’s online experience, 255 (28.4 %) participants agreed and 525 (58.5 %) strongly agreed with the statement that “*they did not experience any technical difficulties with the registration or login process”*. Two hundred and sixty (29.0 %) participants agreed and 546 (60.8 %) strongly agreed that the course webpage and links were easy to understand and navigate.

#### Acceptance of online-learning

Participant’s acceptance of online-learning was evaluated by (1) a preference of the online training in comparison to traditional face-to-face training; and (2) the willingness to recommend the course to other people. Two hundred and forty-nine (27.7 %) participants agreed and 588 (65.5 %) strongly agreed that this online training approach was more effective than traditional face-to-face training. Only 10 (1.1 %) did not agree and 48 (5.3 %) were unsure if the online training course was more effective than traditional training. Among the participants, 258 (28.7 %) would recommend and 577 (64.3 %) would strongly recommend the course to other healthcare workers. Only 54 (6.0 %) were unsure, and 4 (0.4 %) would not recommend the course.

#### Open-ended comments from participants

The open-ended questions at the end of the feedback questionnaire invited participants to provide comments on the positive and negative aspects of the online course of which they observed. In general, a majority of participants expressed their enthusiasm for this new means of training. Examples of positive comments included: its flexibility and time saving advantages in comparison to face-to-face training, and the innovative and interactive design that the course featured. Some participants indicated that they did not like the pre-designated learning process as it restricted them from skipping one section without completing it. Many participants pointed out that they would like to learn more about the treatment plans and medications to treat chronic HBV infection.

## Discussion

The evaluation results of our online training course showed improved HBV prevention and care knowledge among participating health professionals upon completion of the course. Out of a total score of 6, the mean score increased from 3.67 on the pre-course knowledge test to 4.92 on the post- course test. The pre-course knowledge test also confirmed knowledge gap on prevention and care of HBV infected persons among participants: only 50.4 % identified all the transmission routes correctly, and 40.7 % recognized the recommended monitoring tests for individuals who are chronically infected. Our results were consistent with the results of previous studies that demonstrated hepatitis B knowledge gap and training needs of health professionals in China [7, 9, 10].

Our results showed high level of acceptance of the “KnowHBV” online training course as a means to improve HBV prevention and control knowledge among participants. About 93 % of the participants indicated they preferred this course over traditional face-to-face education. Rapid internet infrastructure development within the health system in China made it possible to use online training to rapidly and effectively improve health professionals' knowledge and practice. Compared with traditional face-to-face education, internet-based learning has many advantages in delivering knowledge and skills as a means of CME. Many participants pointed out in the open-ended question session that they enjoyed the flexibility and time-saving-features of the online course as they could access the training and earn CME credits at their working station or at home amidst a busy schedule. Other studies revealed similar comments from participants on the flexibility and convenience of web-based courses to facilitate individualized study pace and schedule [19]. Online learning is also efficient to deliver knowledge to a large number of healthcare practitioners at the same time and reach out to those working in remote and rural areas with significant reduction in both time and money spent compared to the traditional face-to-face and classroom style education [20–22]. This is extremely important for developing countries, where resources are often limited to improve the quality of health care services.

Content quality was observed as the most essential standard for online CME, and previous studies found that evidence-based and needs-driven content generated greater improvements in medical knowledge and quality of service [23, 24]. Our learner-feedbacks results showed that about 93 % of the participants rated the course’s overall quality as ‘good’ or ‘very good’; 96 % reported the course content was useful for their daily work and life, and over 58 % reported that at least half of the content presented was unknown to them, which indicated that the course content was appropriate and relevant in addressing existing knowledge gap.

Our results also demonstrated a potential constraint of online training—low enrollment rate, which was also revealed by Fleisher and colleagues who reported only 24.6 % of the target women in their study logged onto the website and most of them (81 %) logged only once [25]. The enrollment rate for our online course was 29.9 % among the target health professionals working at the selected project sites with concerted communication efforts as well as CME credits as incentive. As such, further research in this regards, which tap into country-specific situations, will contribute to the long-term success of online trainings.

There were a few limitations in our study: the training and surveys were all completed online, and therefore we were unable to monitor the environmental factors that might confound the increased knowledge during the study period. As in all internet assisted tests and surveys, we had no control on how participants completed the online surveys and if they sought outside support before choosing the answers. Since our six questions were quite specific to the course content, and we had a large number of registered participants, we considered that our results were valid. Moreover, we were limited to conclude the effectiveness of “KnowHBV” in short-term knowledge improvement, because our study was unable to follow-up with participants after the completion of the course. Thus, future studies will help to assess the course’s long-term impact in knowledge retention and practice change. Due to the voluntary participation during user recruiting process, participants’ representativeness could be compromised. Since our results on baseline knowledge of the participants were consistent with the vast available data on the knowledge level of Chinese health workers of different demographic categories [7, 9, 10], we concluded that the participants in this study were representative to the health professionals in China. We expect significant hepatitis B prevention and care knowledge improvements among the health professionals in Gansu and Qinghai Province in our province-wide training projects using our free online training course following this evaluation study in Shandong.

## Conclusion

To our knowledge, the “KnowHBV” online training course is the first open-access online course offered to improve HBV infection prevention and care knowledge of health care and public health professionals in China. Our study confirmed that gaps remain in HBV knowledge in transmission routes and long-term care of chronically infected persons. Evaluation results showed that the course was effective in improving knowledge and was well-received and accepted by health professionals in China.

## Abbreviations

CDC: Centers for Disease Control and Prevention
CME: Continuing Medical Education
HBV: hepatitis B virus
